# A Nationwide Analysis of Diabetes Mellitus and Intracranial Injuries: No Impact on Mortality but Prolonged Hospital Stays in Germany

**DOI:** 10.3390/medicina61122187

**Published:** 2025-12-10

**Authors:** Theresia Sarabhai, Lavinia Jürgens, Karel Kostev

**Affiliations:** 1 Department of Endocrinology, Diabetology and Metabolism, Medical Faculty and University Hospital Essen, University Duisburg-Essen, 45147 Essen, Germany; 2 Department of Trauma Surgery and Orthopedics, Hospital Neukölln, 12351 Berlin, Germany; laviniajurgens@gmail.com; 3 Epidemiology, IQVIA, Unterschweinstiege, 60549 Frankfurt am Main, Germany; karel.kostev@iqvia.com; 4 University Clinic, Philipps University, 35043 Marburg, Germany

**Keywords:** diabetes mellitus, intracranial injury, in-hospital mortality, rehospitalization, retrospective cohort study

## Abstract

*Background and Objectives:* Diabetes mellitus (DM) is a growing global health concern linked to increased hospitalization rates, longer hospital stays, and higher mortality. Older adults with DM are particularly prone to intracranial injuries due to frailty and DM-related complications such as neuropathy and cardiovascular diseases. This study explores the impact of DM on in-hospital outcomes in patients with intracranial injuries. *Material and Methods:* This retrospective cohort study used data from 45 hospitals in Germany, including 12,720 patients aged ≥40 years hospitalized between January 2019 and December 2023 with a primary diagnosis of intracranial injury. Patients were categorized based on the secondary presence of DM diagnosis (ICD-10 E10-E14). Outcomes included in-hospital mortality, rehospitalization within 1 year and hospital length of stay (LOS). Multivariable logistic regression models were used to analyze associations between DM and the different outcomes, adjusting for age, sex, hospitalization year, and comorbidities. *Results:* Among 12,720 patients, 2394 had a known DM diagnosis. The median age was higher in DM patients (82 vs. 79 years). In-hospital mortality rates were similar for patients with and without DM (4.7% vs. 4.6%; OR: 0.98; 95% CI: 0.78–1.22). DM was not associated with rehospitalization risk (OR: 1.06; 95% CI: 0.89–1.26) but showed a trend toward longer hospital stays (≥7 days: OR: 1.13; 95% CI: 1.01–1.26). *Conclusions:* While DM did not significantly influence mortality or rehospitalization after intracranial injuries, it showed a non-significant trend towards extended LOS (≥7 days). These findings underscore the importance of targeted management strategies to optimize outcomes in this population.

## 1. Introduction

Diabetes mellitus (DM), especially type-2 DM, poses a growing challenge to global health systems. The global prevalence of DM has risen continuously over recent decades, with estimates indicating that more than 10% of the adult population is affected [1]. The DM prevalence is expected to rise even further in the coming years. This metabolic disorder substantially contributes to morbidity, mortality and healthcare utilization due to its associated complications and comorbidities [2]. Patients with DM experience higher hospitalization rates and more frequent prolonged hospital stays compared to individuals without DM [3]. These heightened hospitalization rates often result from both the acute metabolic complications of DM and the exacerbation of other medical conditions [4].

Intracranial injuries, most commonly resulting from falls, disproportionately affect older adults due to age-related frailty, comorbidities, and impaired mobility [5,6]. DM may further increase this risk, as it often co-occurs with cardiovascular disease, chronic kidney disease, dementia, brain injury and neuropathy [2]. Those associated chronic diseases all contribute to the risk of falling and vulnerability to intracranial injury [5,7]. Several mechanistic pathways, such as hyperglycemia-driven inflammation, impaired wound healing, and increased oxidative stress, have been proposed to influence trauma recovery [7,8]. However, evidence regarding their clinical relevance varies, and some mechanisms remain conceptual rather than empirically validated.

Previous research, primarily from trauma-center cohorts, has reported higher mortality rates among individuals with DM and intracranial injuries, particularly in the presence of stress-induced hyperglycemia [9,10,11]. But the generalizability of these findings to broader hospital populations remains uncertain. Despite the rising prevalence of DM and the high burden of intracranial injuries among older adults, large population-based data from European healthcare systems on DM and in-hospital outcomes remain scarce, especially for older adults [7,12].

Therefore, the aim of this large multicenter retrospective cohort study is to investigate whether DM is associated with (i) in-hospital mortality, (ii) rehospitalization rates, and (iii) hospital length of stay in patients hospitalized for intracranial injuries over a five-year period in Germany. In addition, this study will examine demographic characteristics and DM-associated comorbidities, which are included as covariates in our analytical approach to provide a comprehensive understanding of the interaction between DM and intracranial injury prognosis.

## 2. Methods

### 2.1. Data Source

This multicenter retrospective cohort study utilized data from the hospital database managed by IQVIA^®^ (Frankfurt, Germany). The database comprises standardized data from 46 hospitals across Germany, transmitted to the Reimbursement Institute for Hospitals (InEK) under §21 of the Hospital Compensation Act (KHEntgG). For data protection, export files generated by the software are anonymized by removing case and patient identifiers prior to transmission. Individual treatment episodes included in the §21 dataset are grouped using specialized grouper software developed by 3M Health Information Systems Salt Lake City, UT, USA) and IQVIA^®^. This database has been validated in previous studies investigating in-hospital mortality [13,14], demonstrating high completeness of mandatory variables, consistent ICD-10 coding accuracy, and reliable capture of in-hospital outcomes.

### 2.2. Study Population

This retrospective cohort study included hospitalized patients aged ≥40 admitted between January 2019 and December 2023 with a primary diagnosis of intracranial injury (ICD-10: S06, including subtypes, e.g., concussions, traumatic cerebral edema, diffuse and focal traumatic brain injuries). Patients were classified as having or not having DM (ICD-10: E10-E14) based on their secondary diagnoses. For patients with multiple hospitalizations during the study period, only the first hospitalization was analyzed to avoid redundancy in re-hospitalization data. Covariates were selected a priori based on clinical relevance and previous literature indicating their association with outcomes in diabetes and intracranial injury populations.

### 2.3. Study Outcome and Covariables

The study evaluated in-hospital mortality rates, rehospitalization rates due to intracranial injuries, and hospital length of stay (LOS) in relation to DM. Rehospitalization was defined as any hospital admission occurring within one year after the index event with a primary diagnosis of intracranial injury (ICD-10 S06). External cause codes were not evaluated. Mortality rates were calculated separately for men and women and stratified by age groups. All variables included in the analyses (age, sex, diabetes status, primary diagnosis, outcomes, and comorbidities) were complete in the exported dataset; therefore, no imputation or missing data procedures were required.

### 2.4. Statistical Analyses

Multivariable logistic regression models were used to analyze the associations between DM and in-hospital mortality, rehospitalization rates, and different LOS categories (≥7 days and ≥14 days). As LOS showed a highly right-skewed distribution, LOS was analyzed as a dichotomous variable using two clinically meaningful thresholds (≥7 days and ≥14 days). These cut-offs represent prolonged and very prolonged hospitalizations. These models were adjusted for age, sex, hospitalization year, and secondary diagnoses, including dyslipidemia, hypertension, ischemic heart disease, heart failure, chronic kidney disease, dementia, and alcohol-related disorders. Analyses were conducted for the entire population and stratified by age and sex. Results were presented as odds ratios (ORs) with 95% confidence intervals (CIs). Given the multiple regression models conducted across several outcomes and stratified analyses, we applied a conservative significance threshold of *p* < 0.01 to reduce the likelihood of type I error inflation. The analysis is exploratory in nature, and therefore, no formal correction, such as Bonferroni adjustment, was applied. All analyses were performed using SAS 9.4 (SAS Institute, Cary, NC, USA). Multicollinearity was assessed using variance inflation factors (VIFs), and no problematic collinearity was detected.

## 3. Results

### 3.1. Baseline Characteristics

The present study included 12,720 patients hospitalized with intracranial injury, of which 2394 had DM and 10,326 did not have DM (Figure S1). The median age was higher among patients with DM (82 vs. 79 years). Men constituted 51% of the DM cohort and 49% of the non-DM cohort. All co-diagnoses: dyslipidemia, hypertension, ischemic heart diseases, heart failure, chronic kidney disease, all-cause dementia, except alcohol-related disorders, were more prevalent in the DM cohort than in the without DM cohort (Table 1).

### 3.2. Association Between Diabetes and In-Hospital Mortality

**Figure 1:**
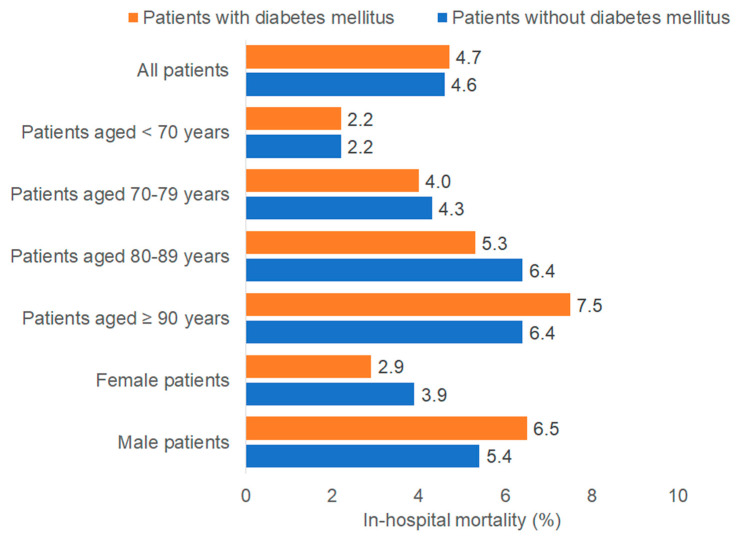
In-hospital mortality in intracranial injury patients with and without diabetes mellitus. Bar chart displaying the proportion of patients who died during hospitalization for intracranial injury, stratified by diabetes status (orange = with diabetes mellitus; blue = without diabetes mellitus). Mortality percentages are shown for the total cohort (DM: n = 2394; non-DM: n = 10,326) and across age groups (<70, 70–79, 80–89, ≥90 years) and sex (female, male). Overall, mortality rates were similar between patients with and without diabetes across all strata.

The mortality rates by DM status are depicted in Figure 1. Overall, in-hospital mortality was similar in patients with and without DM (4.7% vs. 4.6%). In the multivariable regression model, DM was not associated with in-hospital mortality (OR: 0.98; 95% CI: 0.78–1.22) regardless of the sex or age group (Table 2).

### 3.3. Association Between Diabetes and Rehospitalization Rate

**Figure 2:**
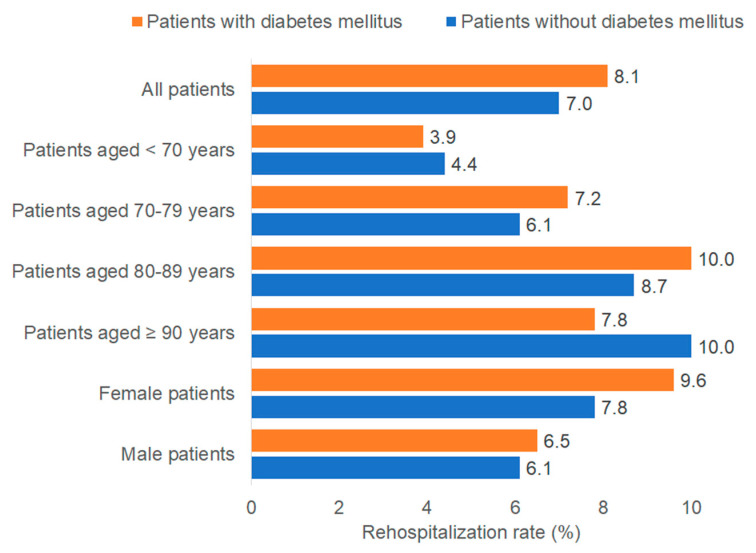
One-year rehospitalization rates in intracranial injury patients with and without diabetes mellitus. Bar chart showing the percentage of patients who were rehospitalized within one year with a primary diagnosis of intracranial injury, stratified by diabetes status (orange = with diabetes mellitus; blue = without diabetes mellitus) and by demographic subgroup. The total cohort included n = 2394 patients with diabetes mellitus and n = 10,326 patients without diabetes mellitus. Rehospitalization percentages for each subgroup are displayed.

The rehospitalization rates by diabetes status are depicted in Figure 2. A total of 8.1% of patients with DM and 7.0% without DM were rehospitalized for intracranial injuries. However, multivariable regression analysis revealed no significant association between DM and rehospitalization rate (OR: 1.06; 95% CI: 0.89–1.26). This finding remained consistent across age and sex stratifications (Table 2).

### 3.4. Association Between Diabetes and Hospital Length of Stay

**Figure 3:**
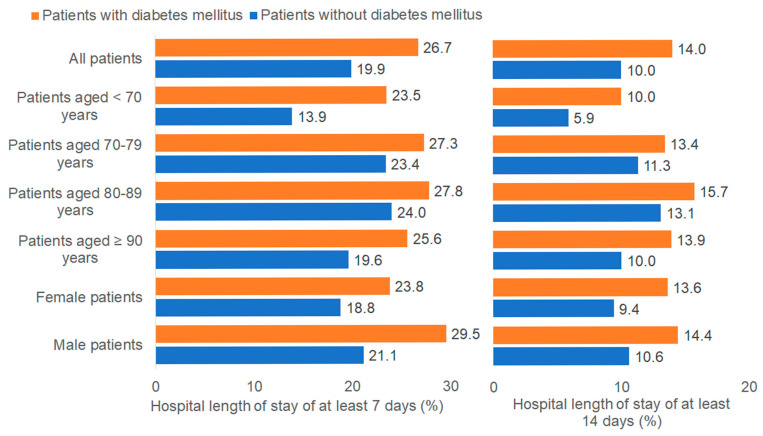
Proportion of intracranial injury patients with and without diabetes mellitus with a hospital length of stay of at least 7 and 14 days. Bar charts show the percentage of patients hospitalized for intracranial injury who remained in the hospital for ≥7 days (**left panel**) and ≥14 days (**right panel**), stratified by diabetes status (orange = with diabetes mellitus; blue = without diabetes mellitus) and by demographic subgroup. The total cohort included n = 2394 patients with diabetes mellitus and n = 10,326 patients without diabetes mellitus. Percentages for each subgroup are displayed.

The LOS with and without diabetes mellitus are depicted in Figure 3. The median LOS was 2 days (IQR: 1–7 days) for patients with DM and 2 days (IQR: 1–5 days) for those without DM. A higher proportion of patients with DM had extended hospital stays: 26.7% stayed for ≥7 days, and 14.0% stayed for ≥14 days, compared to 19.9% and 10.0% without DM, respectively. Multivariable regression analysis showed that DM was not significantly associated with an LOS of ≥7 days (OR: 1.13; 95% CI: 1.01–1.26) or ≥14 days (OR: 1.09; 95% CI: 0.95–1.25) (Table 2).

## 4. Discussion

This study aimed to evaluate the impact of DM on in-hospital mortality, rehospitalization rates, and hospital length of stay among patients hospitalized with intracranial injuries with and without a known diabetes mellitus diagnosis. Using a large multicenter dataset from German hospitals, we found that DM was not significantly associated with in-hospital mortality or rehospitalization rates. However, DM showed a trend toward an increase in hospital length, likely due to the higher prevalence of metabolic disturbances characteristic of DM, diabetes-associated comorbidities, and a greater incidence of diabetes-related complications in these patients. To our knowledge, this is the first nationwide retrospective cohort study in Germany to evaluate hospital metrics among intracranial injury patients with and without DM.

This study found no significant difference in in-hospital mortality rates between patients with and without diabetes mellitus (DM) hospitalized for intracranial injuries, regardless of sex or age group. This finding is particularly remarkable given the significantly higher prevalence of comorbidities among the diabetic cohort, such as hypertension, chronic kidney disease, and ischemic heart disease—conditions typically associated with worse outcomes and increased mortality in hospitalized patients [2]. Hyperglycemia seems to be a key factor in poor outcomes following traumatic brain injuries, with mortality rates reaching 14% in patients with DM compared to 8.2% in non-diabetics [9,15,16]. Insulin-dependent diabetes mellitus further increases this risk, with mortality rates as high as 17.1% compared to non-insulin-dependent diabetes mellitus [17]. Findings from a large Taiwanese population-based study suggest that stress-induced hyperglycemia, rather than diabetic hyperglycemia, is the more significant predictor of in-hospital mortality in moderate to severe intracranial injury patients, which indicates distinct mechanisms between chronic diabetic and stress-induced hyperglycemia [16]. Also, patients with severe intracranial injuries seem to exhibit significantly higher serum glucose levels than those with mild injuries [18]. Further cohort studies indicate that stress-induced hyperglycemia, strongly linked to systemic stress and injury severity, is the main driver of increased mortality in intracranial injuries [16,19,20,21]. This may explain the difference in findings in our study, as we analyzed the comorbidity of diabetes mellitus, rather than hyperglycemia, in patients with intracranial injuries. Similarly, a retrospective analysis of 77 patients with severe brain injuries found no association between DM and mortality rates but identified early hyperglycemia as a significant predictor of in-hospital mortality [22]. Further differences between our findings and earlier reports may partly reflect variation in study populations. Many prior studies were based on trauma-center cohorts with higher injury severity, whereas our population represents a broader spectrum of hospitalized patients with intracranial injuries. The severity of the injury itself may outweigh the influence of DM or its associated complications [22]. These distinctions likely contribute to the divergent mortality patterns observed.

This study found no significant difference in rehospitalization rates within one year between patients with and without DM following an intracranial injury, regardless of age or sex group. This result is surprising, as prior research consistently indicates that patients with DM typically experience higher rates of rehospitalization due to complications such as poor glycemic control and acute diabetic complications [23,24]. A recent study report that diabetes-related hospitalizations have significantly increased, with up to 11.3% of hospitalizations for type 2 DM resulting in higher readmission, especially among younger males aged 18–29 years [25]. Rehospitalization is also a significant burden for intracranial injured patients, with rates as high as 20% for at least three years after injury (Cifu et al., 1999) [26]. However, rehospitalizations appear to be primarily driven by injury-specific complications, such as recurrent subdural hematomas or the need for neurorehabilitation, rather than metabolic disturbances linked to DM [27]. For instance, elderly patients admitted with recurrent subdural hematomas face elevated readmission risks due to injury severity and comorbidities, including DM, highlighting the complex interplay between systemic health and injury outcomes [27]. Additionally, patients recovering from intracranial injuries often receive highly coordinated and comprehensive care plans, including proactive discharge planning and regular follow-up, which may mitigate the potential impact of DM on rehospitalization rates. Such integrated care approaches appear to effectively address both injury-related needs and underlying comorbidities, potentially leveling the rehospitalization risk between diabetic and non-diabetic patients [26]. Furthermore, structural characteristics of the German healthcare system, including standardized post-discharge pathways and coordinated rehabilitation processes, may mitigate rehospitalization risk and contribute to the absence of group differences.

Interestingly, patients with DM demonstrated a trend towards longer lengths of hospital stay for the ≥7-day threshold compared to those without DM after admission for intracranial injury. This finding is consistent with prior studies [8,28]. A prolonged LOS is likely attributable to metabolic disturbances, which are characteristic of DM, such as diabetes-associated comorbidities, exacerbating systemic dysfunction and delaying recovery from trauma [12]. Both hyper- and hypoglycemia interrupt critical cellular processes, worsening secondary brain injuries and increasing the need for intensive care [29]. Notably, a bell-shaped relationship between average blood glucose levels and long-term outcomes, such as the Glasgow Outcome Score, highlights that both excessively high and low blood glucose levels suggest a poor prognosis and prolonged hospital stays in intracranial injury [30]. These metabolic disturbances, compounded by comorbidities, may lead to a cycle of complications, infections, and delayed recovery. The higher risk of falls and subsequent injuries among individuals with DM, particularly those on insulin therapy, may also contribute to longer hospitalizations [31]. In addition to biological mechanisms, non-clinical factors may also contribute to prolonged LOS among patients with DM, including administrative discharge criteria, coordination of rehabilitation placement, and social support availability. Further, in the context of brain injury, extreme hospital length of stay seems to be associated with socioeconomic factors, such as low socioeconomic status and Medicaid insurance, contributing to prolonged hospitalization in the USA [28]. However, population-based data on hospital metrics, particularly length of hospital stay, in patients with intracranial injuries are lacking, making it challenging to thoroughly compare our findings.

A major strength of this study is its use of a large nationwide multicenter dataset, standardized ICD-10 coding across institutions, and inclusion of recent real-world data from 2019–2023, supporting the robustness and generalizability of our findings. The adjustment for demographic and clinical variables further supports the reliability of our conclusions.

Overall, the limitations of this study arise both from the nature of administrative data and from the observational study design, which is susceptible to residual confounding and does not permit causal inference. The dataset lacked detailed information on diabetes management, including glycemic control, diabetes duration, and treatment regimens. Key clinical variables such as Glasgow Coma Scale, radiological severity, diabetes duration, treatment regimen, and glycemic measures (admission glucose, HbA1c, acute hyperglycemia) were unavailable. Then, the study did not include information on the severity of intracranial injuries (e.g., Injury Severity Score, the Abbreviated Injury Score or Glasgow Coma Scale) or the presence of other acute complications at admission. These factors may be critical in determining patient prognosis and may influence the observed outcomes. Fourth, the reliance on ICD-10 codes for identifying diagnoses and comorbidities may introduce coding inaccuracies and potentially misclassification of patients and diagnoses. Also, as the dataset does not capture undiagnosed DM, some degree of non-differential misclassification cannot be excluded. Additionally, while the dataset is large and multicenter, it is limited to hospitals in Germany, which may impact the generalizability of the findings to other healthcare systems or populations. Lastly, the study focused on short-term outcomes, such as in-hospital mortality and rehospitalization within one year, without assessing longer-term recovery, functional outcomes, or quality of life. Future studies should aim to address these gaps to provide a better understanding of the impact of diabetes on patients with intracranial injuries.

## 5. Conclusions

In this large multicenter retrospective cohort study of patients hospitalized with intracranial injuries in Germany, a secondary diagnosis of DM was not associated with increased in-hospital mortality or rehospitalization rates, despite the substantially higher burden of comorbidities among individuals with DM. However, DM was associated with a non-significant trend toward prolonged hospital stays at the ≥7-day threshold, which may reflect greater clinical complexity and slower recovery trajectories in this population.

These findings highlight that while DM does not appear to worsen short-term survival following intracranial injury, it may have implications for inpatient resource utilization, including higher care demands and potentially increased hospital costs. The prolonged hospitalization observed in patients with DM likely reflects the cumulative effects of glucose dysregulation, metabolic disturbances, and the higher prevalence of cardiovascular, renal, and neurological comorbidities. Given the rising prevalence of DM and the high incidence of intracranial injuries among older adults, targeted inpatient management strategies, including optimized glycemic control, early identification of high-risk metabolic and cardiovascular profiles, and coordinated multidisciplinary care, may help reduce hospital stays and improve overall outcomes. Future research should prioritize integrating glycemic parameters and detailed injury severity measures to clarify underlying mechanisms of the interplay between chronic diabetes and acute neurological trauma and improve risk stratification.

## Figures and Tables

**Table 1:** Baseline characteristics of the study sample.

	Patients with Diabetes Mellitus (n = 2394)	Patients Without Diabetes Mellitus (n = 10,326)	*p* Value *	Standardized Mean Differences
**Age**				
Median age (IQR))	82 (12)	79 (23)	<0.001	0.375
<70 years, n (%)	409 (17.1)	3457 (33.5)	<0.001	−0.384
70–79 years, n (%)	545 (22.8)	1838 (17.8)		0.125
80–89 years, n (%)	1159 (48.4)	3706 (35.9)		0.255
≥90 years, n (%)	281 (11.7)	1325 (12.8)		−0.034
**Sex, n (%)**				
Female	1184 (49.5)	5350 (51.8)	0.038	−0.046
Male	1210 (50.5)	4976 (48.2)		0.046
**Hospitalization year, n (%)**				
2019	190 (7.9)	842 (8.2)		−0.011
2020	365 (15.3)	1594 (15.4)		−0.003
2021	536 (22.4)	2275 (22.0)	0.465	0.010
2022	609 (25.4)	2468 (23.9)		0.035
2023	694 (29.0)	3147 (30.5)		−0.033
**Co-diagnoses, n (%)**				
Dyslipidemia	601 (25.1)	1239 (12.0)	<0.001	0.383
Hypertension	1803 (75.3)	5112 (49.5)	<0.001	0.553
Ischemic heart diseases	519 (21.7)	955 (9.3)	<0.001	0.348
Heart failure	311 (13.0)	620 (6.0)	<0.001	0.240
Chronic kidney disease	464 (19.4)	709 (6.9)	<0.001	0.376
All-cause-dementia	527 (22.0)	1618 (15.7)	<0.001	0.161
Alcohol related disorders	112 (4.7)	935 (9.1)	<0.001	−0.174

Proportions of patients in N and % given, unless otherwise indicated. IQR- interquartile range. * Wilcoxon signed rank test for age, Chi^2^ test for other variables.

**Table 2:** Association of diabetes mellitus with the in-hospital mortality, rehospitalization and with the in-hospital length of stay of at least 7 and 14 days in patients hospitalized for intracranial injury (multivariable logistic regression).

Variables	In-Hospital Mortality		Rehospitalization Due to Intracranial Injury		In-Hospital Length of Stay of at Least 7 Days		In-Hospital Length of Stay of at Least 14 Days	
	OR (95% CI)	*p* Value	OR (95% CI)	*p* Value	OR (95% CI)	*p* Value	OR (95% CI)	*p* Value
**Total**	0.98 (0.78–1.22)	0.849	1.06 (0.89–1.26)	0.526	1.13 (1.01–1.26)	0.028	1.09 (0.95–1.25)	0.245
**<70 years**	0.87 (0.41–1.85)	0.714	0.69 (0.40–1.21)	0.194	1.25 (0.95–1.64)	0.106	0.97 (0.66–2.43)	0.892
**70–79 years**	1.08 (0.64–1.83)	0.769	1.21 (0.81–1.81)	0.353	1.00 (0.80–1.27)	0.973	0.96 (0.70–1.30)	0.775
**80–89 years**	0.84 (0.62–1.15)	0.277	1.20 (0.95–1.52)	0.118	1.03 (0.88–1.21)	0.705	1.02 (0.84–1.24)	0.819
**≥90 years**	1.29 (0.76–2.18)	0.345	0.69 (0.43–1.12)	0.138	1.29 (0.94–1.76)	0.115	1.27 (0.86–1.89)	0.236
**Female**	0.75 (0.51–1.11)	0.149	1.17 (0.93–1.47)	0.182	1.07 (0.91–1.26)	0.399	1.14 (093–1.39)	0.210
**Male**	1.14 (0.87–1.51)	0.347	0.93 (0.71–1.21)	0.581	1.19 (1.02–1.38)	0.026	1.04 (0.86–1.27)	0.670

## Data Availability

The datasets used and analyzed during the current study are available from the corresponding author on reasonable request.

## Supplementary Materials

The following supporting information can be downloaded at: https://www.mdpi.com/article/10.3390/medicina61122187/s1, Figure S1. Selection of study patients.
