# Supplementary material for: A Nationwide Analysis of Diabetes Mellitus and Intracranial Injuries: No Impact on Mortality but Prolonged Hospital Stays in Germany
Source: Medicina (Kaunas). 2025 Dec 10;61(12):2187. doi: 10.3390/medicina61122187 (PMC12734764; doi:10.3390/medicina61122187)

Supplement Material (A Nationwide Analysis of Diabetes Mellitus and Intracranial Injuries: No Impact on Mortality but Prolonged Hospital Stays in Germany, Sarabhai et al.)

Figure S1. Selection of study patients

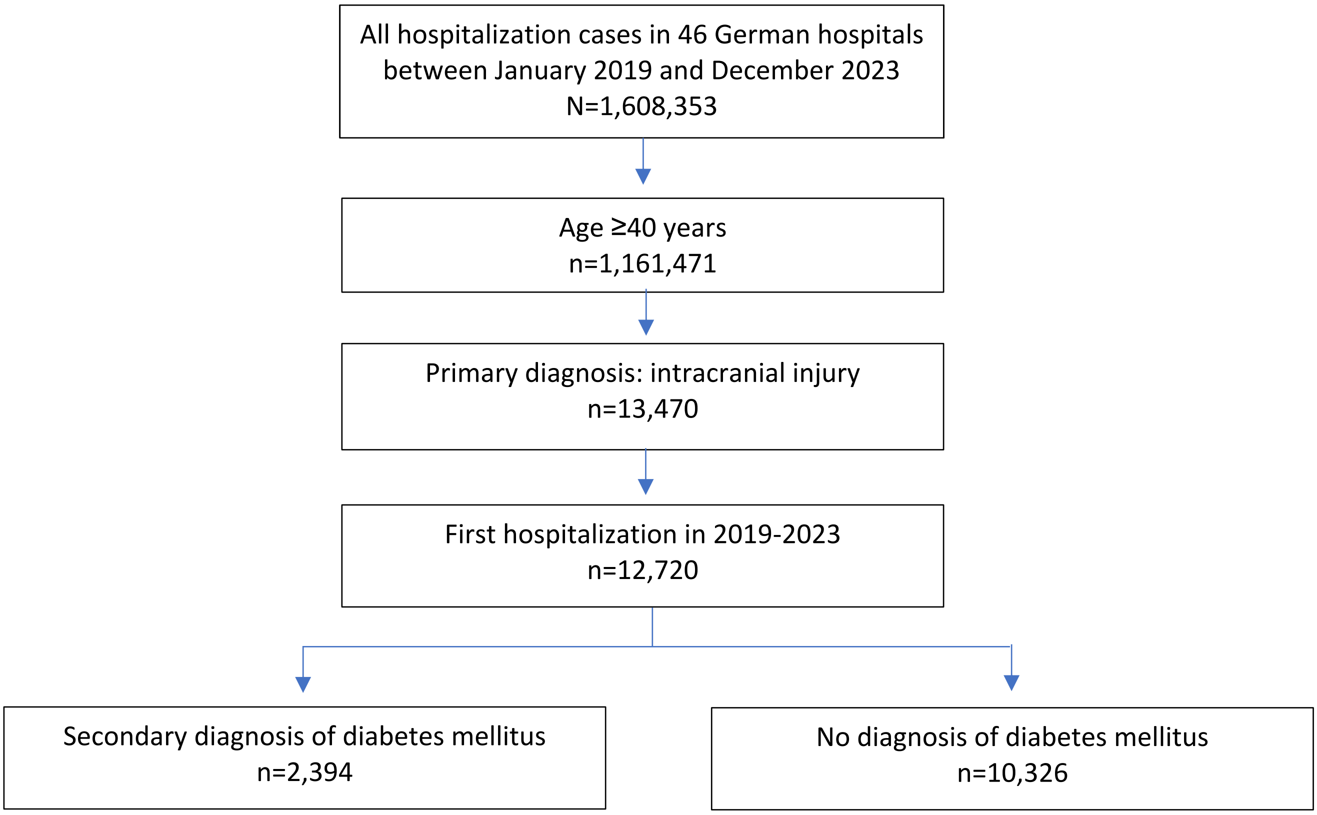

Supplement: Supplementary file 1 [file medicina-61-02187-s001.zip › medicina-4021103-supplementary.pdf]
